# Chronic low-dose exposure to a mixture of environmental endocrine disruptors induces microRNAs/isomiRs deregulation in mouse concomitant with intratesticular estradiol reduction

**DOI:** 10.1038/s41598-017-02752-7

**Published:** 2017-06-13

**Authors:** Julio Buñay, Eduardo Larriba, Ricardo D. Moreno, Jesús del Mazo

**Affiliations:** 1 Department of Physiology, Pontificia Universidad Católica de Chile, Santiago, Chile; 2 Department of Cellular and Molecular Biology, Centro de Investigaciones Biológicas (CSIC), Madrid, Spain

## Abstract

Humans are environmentally exposed not only to single endocrine-disrupting chemicals (EDCs) but to mixtures that affect their reproductive health. In reproductive tissues, microRNAs (miRNAs) are emerging as key targets of EDCs. Here, we analysed changes in the testis “miRNome” (and their biogenesis mechanism) in chronically exposed adult mice to a cocktail of five EDCs containing 0.3 mg/kg-body weight (BW)/day of each phthalate (DEHP, DBP, BBP) and 0.05 mg/kg-BW/day of each alkylphenol (NP, OP), from conception to adulthood. The testis “miRNome” was characterised using next-generation sequencing (NGS). Expression levels of genes involved in miRNA biogenesis were measured by RT-qPCR, as well as several physiological and cytological parameters. We found two up-regulated, and eight down-regulated miRNAs and thirty-six differentially expressed isomiRs along with an over-expression of *Drosha*, *Adar* and *Zcchc11*. A significant decrease of intratesticular estradiol but not testosterone was detected. Functional analysis showed altered spermatogenesis, germ cell apoptosis and negative correlation of *miR-18a-5p* with *Nr1h2* involved in the deregulation of the steroidogenesis pathway. Here, we present the first association between miRNA/isomiRs deregulation, their mechanisms of biogenesis and histopathological and hormonal alterations in testes of adult mice exposed to a mixture of low-dose EDCs, which can play a role in male infertility.

## Introduction

Endocrine-disrupting chemicals (EDCs) have been described as “an exogenous substance or mixture that alters function(s) of the endocrine system and consequently causes adverse health effects in an intact organism, or its progeny, or (sub) populations”^1, 2^. Hundreds of compounds have been considered potential EDCs but only recently, scientific criteria have been established to determine its effects and legislate its use^3, 4^. Male reproductive systems, in particular, are reported to be a relevant target of EDCs^5^.

EDCs vary in its chemical nature and mechanisms of action. In fact, defined profiles of deregulation including transcriptome patterns have been associated with exposure to a single EDC^6^, despite environmental exposure involving the intake of mixtures of different compounds. Since most studies on EDCs effects are done on a single compound, an underestimation of the combined EDCs risk has emerged as an exposure to mixtures is currently prevalent, generally at low doses^7^.

Alkylphenols and phthalates are common EDCs that industries use for the manufacture of a wide range of products, such as bottles, food packaging, personal care products, and cleaners. The most commonly used alkylphenols are nonylphenol (NP) and octylphenol (OP) while six phthalates are present in consumer products: di-(2-ethylhexyl) phthalate (DEHP), diisononyl phthalate (DINP), dibutyl phthalate (DBP), diisodecyl phthalate (DIDP), di-n-octyl phthalate (DnOP), and benzyl butyl phthalate (BBP or BzBP). In spite of the fact that these compounds can be combined to give plastic different properties, their leakage from the containers made of this material are a concern to the environment^8, 9^. Thus, humans are chronically exposed to low-doses of combinations of alkylphenols and phthalates throughout their lives. To support this statement, there are data showing that these chemicals are present in human samples such as blood, breast milk, urine, amniotic fluid, and semen^10^, and even in complex developmental organs such as the placenta^11^. Alkylphenols and phthalates are considered EDCs since *in vivo* studies have shown that exposure (individual or in mixture) to them correlates with changes in reproductive hormones levels such as testosterone and/or estradiol along with transcriptional modifications of genes that encode proteins involved in the steroidogenic pathway such as *Star*, *Cyp11a1*, *Cyp17a1*, *Hsd3b1*, *Cyp19a1* and transcriptional factors controlling those genes (e.g. *Sp1*). Thus, to unveil the molecular mechanism of endocrine disruption it is necessary to understand how alkylphenols and phthalates deregulate the gene expression of the steroidogenic pathway.

The effects of phthalates and alkylphenols at doses below acute toxicity could not only be due to a deregulation at the transcriptional level but also to changes in the fine regulatory post-transcriptional systems that mediate them. MicroRNAs (miRNAs) are small, endogenous non-coding RNAs (ncRNAs), usually 20–25 nucleotides long and evolutionarily well-conserved across metazoans^12^. They comprise a mechanism of negative regulation of gene expression in a sequence-specific manner that is present in all cells and developmental processes and could play a part in diverse pathologies. Although some aspects of their processing are still poorly understood, most of their basic biogenesis including the canonical and functional variants (isomiRs), is well established^13, 14^.

In testes, genetic ablation of *Drosha* or *Dicer* (two gene-encoding enzymes involved in miRNAs biogenesis) in Sertoli and germ cells leads to a severe impairment of spermatogenesis^15, 16^ and a serious deregulation of miRNAs processing and gene expression. In humans, certain male reproductive dysfunctions are associated with the aberrant expression of specific miRNAs^17, 18^.

The effects of various EDCs on the deregulation of some miRNAs and consequently on its mRNAs targets have already been studied. Evidence *in vitro* ^19, 20^ and *in vivo* ^21^ indicates that exposure to a single EDC can deregulate the expression of specific miRNAs. However, there has been no assessment of the outcomes of an exposure to a mixture of EDCs commonly present in the environment, such as phthalates and alkylphenols, on the ‘miRNome’ or on the miRNAs biogenesis in testes.

Therefore, our general aim was to determine the consequences of a chronic exposure to a mixture of phthalates and alkylphenols on the testes of male mice and in particular to study the changes in the expression pattern of miRNA/isomiRs which act as regulators of gene expression in testes. We also assessed testis damage and changes in the genes responsible for encoding proteins that are involved in the biogenesis, processing, editing, stability, or degradation of miRNAs.

## Results

### Exposure to a mixture of EDCs changed the testes histology and increased germ cell apoptosis

**Figure 1:**
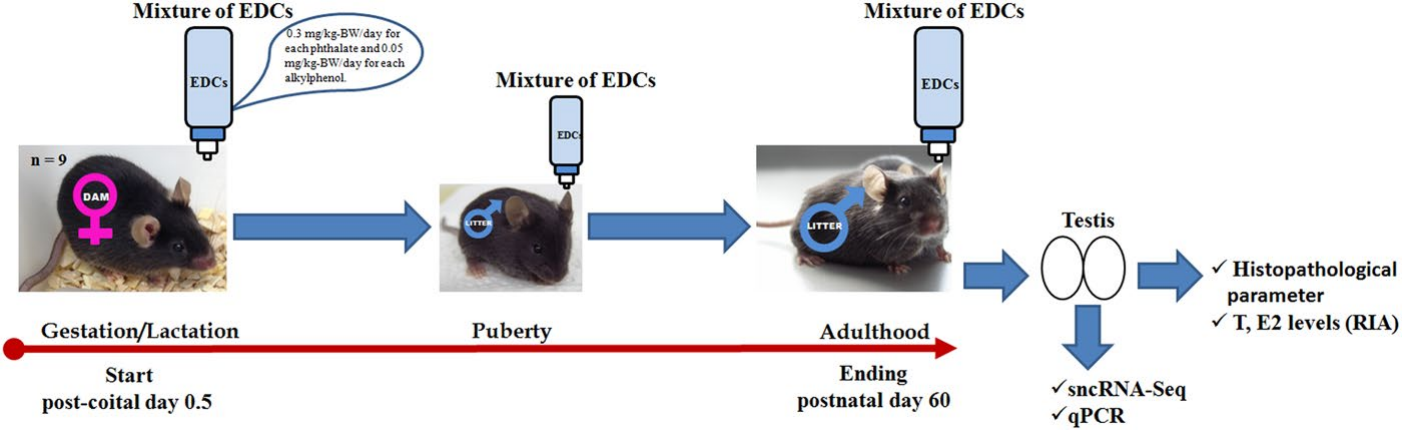
Schematic diagram of the experimental design. C57BL/6J mice were treated as described in the material and methods section. Mice chronically exposed to EDCs-mixture and control mice were sacrificed on postnatal day 60 at which testes were harvested. We quantified intratesticular testosterone and estradiol levels by RIA, histological alterations, expression of mRNAs by RT-qPCR and miRNAs/isomiRs expression by sncRNA-Seq and RT-qPCR.

**Figure 2:**
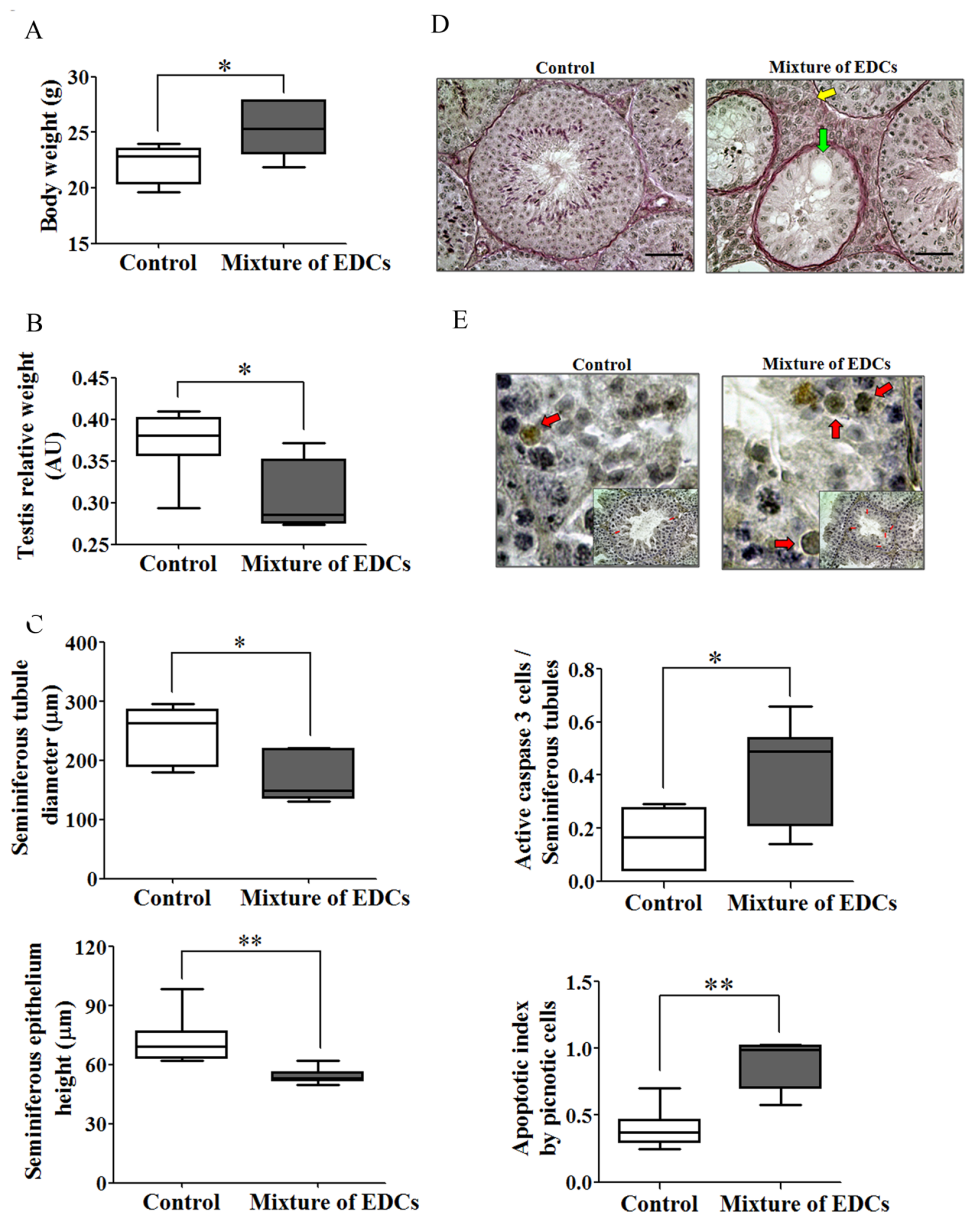
Testes injury and germ cell apoptosis in mice exposed to the mixture of EDCs. (**A**) Mouse body weight. (**B**) Relative weight of testis. (**C**) Morphometric analysis of seminiferous tubules (diameter and epithelium height). (**D**) Representative picture of PAS/hematoxylin staining of seminiferous tubules, yellow arrow shows Leydig cell lesion (hypertrophic/hyperplastic) and green arrow shows seminiferous tubule degeneration. (**E**) Representative pictures of caspase-3 positive germ cells (red arrow) in seminiferous tubules and quantification of the number of caspase-3 positive germ cells and pyknotic cells. All graphics represent the mean ± SEM. For A-B) control group n = 9, EDCs mixture exposed group n = 6. Abbreviation: AU, arbitrary units. (**C**) Control and EDCs mixture exposed group n = 6, Mann–Whitney U test, *p < 0.05; **p < 0.01. (**D–E**) Control group n = 4, EDCs mixture exposed group n = 3 (100 seminiferous tubules), Mann–Whitney U test, *p < 0.05, **p < 0.01.

Adult male mice exposed to a mixture of phthalates and alkylphenols (Fig. 1) presented higher body weight when compared to control mice. However, testis relative weight, diameter and epithelium height of seminiferous tubules was lower in exposed mice (Fig. 2A–C). Regarding testis histology, we observed that exposed mice presented degeneration of seminiferous tubules and hypertrophy/hyperplasia in some areas of the Leydig cells (Fig. 2D, arrows). In addition, exposed animals presented an increase of seminiferous tubules with germ cells exfoliated towards the tubular lumen and tubules without lumina together with a decrease of the frequency of stages VI–VII of the seminiferous epithelium cycle. Moreover, a significant number of seminiferous tubules could not be assigned to any specific stage (see Supplementary Fig. S1). Previous studies have demonstrated that some of the EDCs used in this work, when administered individually, induced germ cell apoptosis in male rats^22^. We show here that the number of pyknotic cells and caspase-3 positive cells, significantly increased in the testes of exposed animals compared to control animals (Fig. 2E,F).

**Figure 3:**
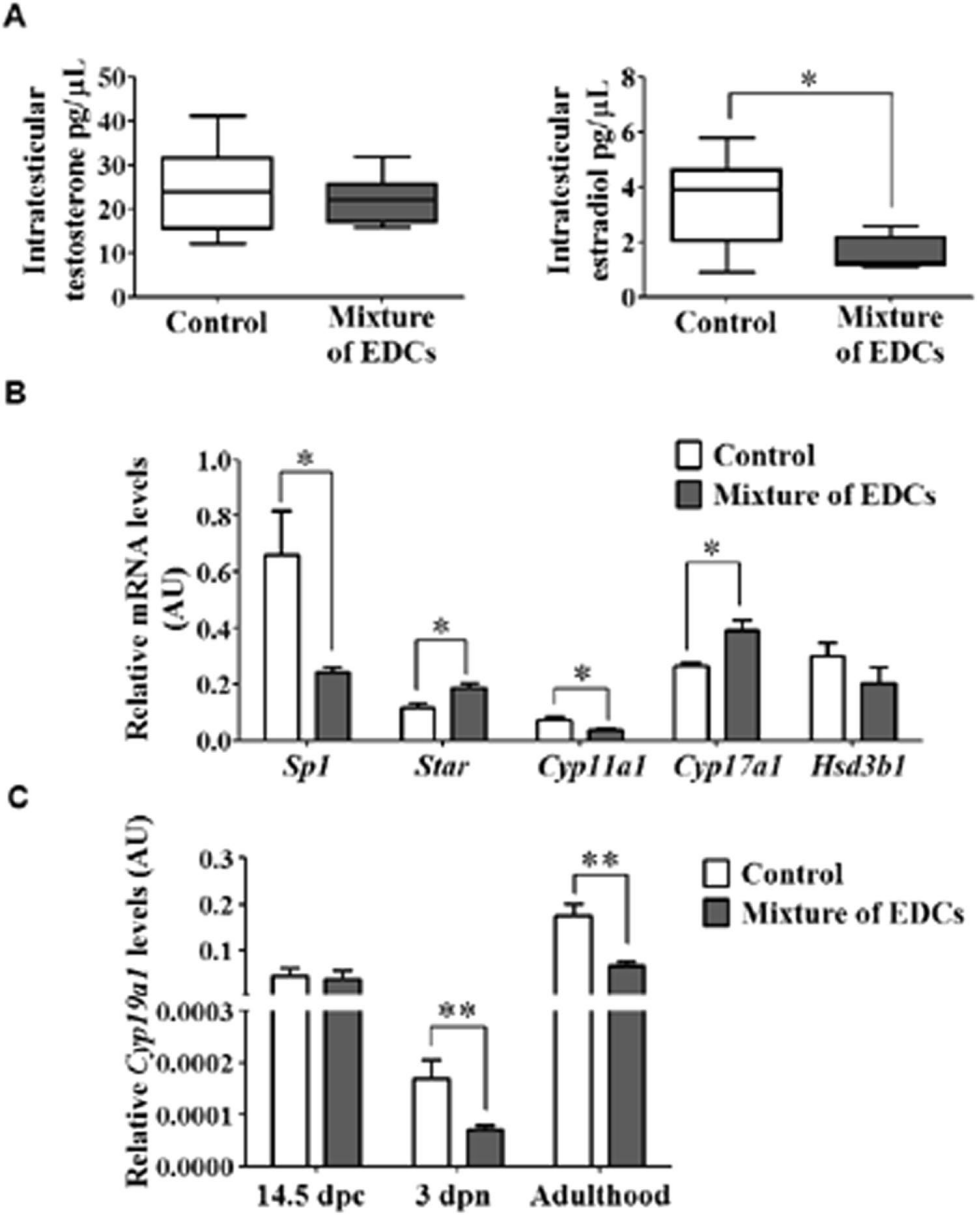
Steroidogenic-pathway enzyme deregulation and estradiol decrease in mice exposed to the mixture of EDCs. (**A**) Testosterone and estradiol levels of seminiferous tubular fluid were measured by RIA, two individual measures per testis (right and left) run in duplicate. (**B**) RT-qPCR of genes implicated in the steroidogenic process. (**C**) *Cyp19a1* expression levels in embryonic gonads of male mice on post-coital day (dpc) 14.5, postnatal day (dpn) 3, and adult mouse testes at postnatal day 60. All graphics represent the mean ± SEM, n = 4. Unpaired t test, *p < 0.05, **p < 0.01. Abbreviation: AU, arbitrary units.

Furthermore, exposed mice showed a decrease in intratesticular estradiol levels but not in testosterone levels (Fig. 3A), which suggested deregulation of genes involved in the biosynthesis of these hormones, such as the transcription factor *Sp1*, cholesterol transporter to the mitochondria (*Star*), and/or enzymes of the steroidogenic pathway (*Cyp11a1*, *Cyp17a1*, *Hsd3b1*, *Cyp19a1*). Using RT-qPCR, we quantified the mRNA levels of these genes, in control and exposed mouse testes. The mRNA levels of *Star* and *Cyp17a1* were up-regulated in exposed animals, whilst those of *Sp1* and *Cyp11a1* were down-regulated (Fig. 3B). There were no significant differences in *Hsd3b1* mRNA expression (Fig. 3B). Since the enzyme aromatase (Cyp19a1) is involved in estradiol synthesis from testosterone, and male reproductive disorders may originate in foetal life or childhood, we assessed the expression of *Cyp19a1* in different periods of gonadal development in control and exposed mice. Besides adult animals, we measured *Cyp19a1* on: 1) post-coital day (dpc) 14.5, when steroid production in testis begins; 2) postnatal day (dpn) 3, when steroid production reaches low levels and 3) adulthood. We found that *Cyp19a1* expression in testes of exposed mice decreased during neonatal development and adulthood, but no change in early embryonic gonads development was detected (Fig. 3C).

Experimental data showed that chronic exposure to a mixture of five different compounds (DEHP, DBP, BBP, NP, and OP) induced significant hormonal and histological alterations in mouse testes, particularly in the expression of genes that encode proteins involved in steroidogenesis. Also, significant increase in the proportion of apoptotic germ cells was observed. This supports the hypothesis that this cocktail of compounds behaves as an endocrine disruptor in male mice.

### A mixture of EDCs changes mRNA levels of genes involved in the biogenesis, editing, and stability of miRNAs

Previous studies have shown that miRNAs are important to testes biology since genetic ablation of *Dicer* or *Drosha* impairs its development and normal spermatogenesis^15, 16^. We wondered whether exposure to the mixture of EDCs could affect miRNAs biogenesis and function. To answer this question, we quantified the mRNAs expression levels of genes that encode proteins involved in pri-miRNAs processing (*Drosha*), nuclear export (*Xpo5*), stability/degradation (*Lin28, Zcchc11, Zcchc6, and Snd1*), editing (*Adar*) and processing of pre-miRNAs (*Dicer*, and *Ago2*).

**Figure 4:**
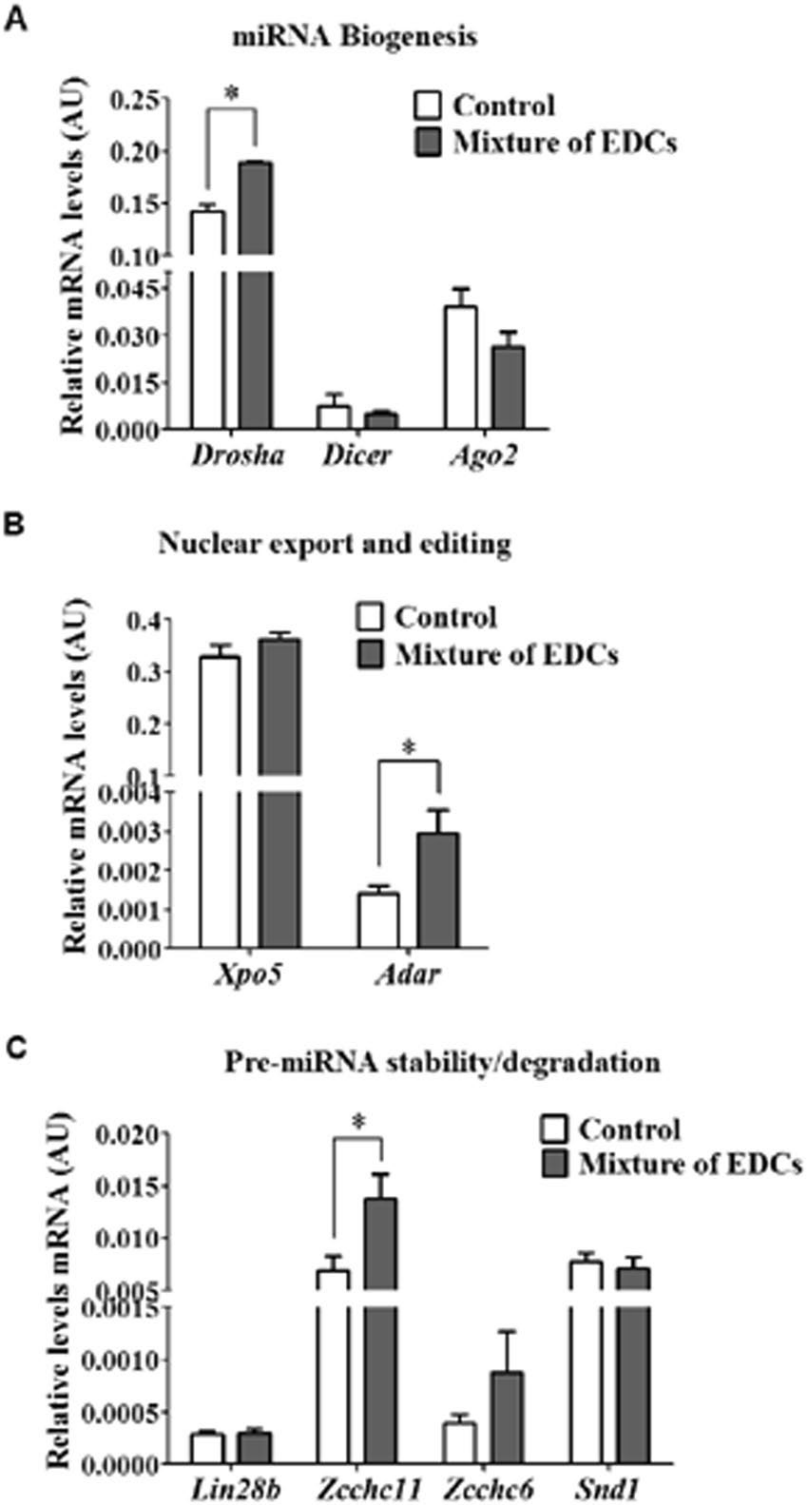
Exposure to the mixture of EDCs induces changes in the expression of genes that encode proteins involved in miRNAs biogenesis and function. RT-qPCR analysis of mRNA levels of genes that encode proteins involved in: (**A**) miRNAs biogenesis, (**B**) miRNAs nuclear export and editing, (**C**) pre-miRNA stability. Values represent the mean ± SEM, Mann–Whitney U-test, n = 3, *p < 0.05. Abbreviation: AU, arbitrary units.

We found significantly increased levels of *Drosha* and *Adar* mRNA in testes of exposed mice (Fig. 4A,B) and levels of *Zcchc11* mRNA, but not *Zcchc6*, two-fold higher than those found in control mice (Fig. 4C). mRNA levels of *Dicer*, *Xpo5*, *Ago2*, *Lin28b*, and *Snd1* were similar to those of control mice (Fig. 4A,B,C).

These results suggest that exposure to a mixture of EDCs could affect the levels of some mRNAs which encode enzymes implicated in pri-miRNAs processing (*Drosha*), editing (*Adar*) and pre-miRNAs stability/degradation (*Zcchc11*), therefore promoting imbalance in the miRNA processing machinery and altering some miRNA functional levels. It could also point out to a novel mechanism of toxic stress response in the testes as a consequence of EDCs exposure.

### Exposure to an EDCs mixture changes the expression of a small group of miRNAs and isomiRs

Since we found out that some genes that encode proteins involved in the biogenesis and processing of miRNAs were deregulated in the testes of mice exposed to the EDCs mixture, we decided to perform next-generation sequencing (NGS) of sncRNA to analyse the miRNome in both exposed and control mice. After trimming and cleaning the sncRNA reads, we mapped them against the mouse genome. Using the miRNA genome coordinates from miRBase v21, we assigned the sncRNA sequences that were mapped into the categories: “precursor-miRNAs”, “canonical mature miRNAs”, and “non-canonical forms or isomiRs”.

Out of the 1193 miRNAs precursors and 1915 mature miRNAs presented in the mouse miRBase v21, we were able to detect 540 miRNAs precursors and 772 canonical mature miRNAs in samples of control mice, and 534 miRNAs precursors and 761 canonical mature miRNAs in samples of ECDs exposed mice (Table 1). Overall, the data showed that 0.75% of pre-miRNAs and 1.31% of canonical miRNAs (ten mature miRNAs) were differentially expressed in control and exposed mice (Table 1). These results suggested that small changes in the miRNome could induce alterations in the phenotypes of testes due to a chronic exposure to the mixture of EDCs.

**Table 1:** Global analysis of miRNAs population in testes of control mice and EDCs mixture-exposed mice.

ID	No. of detected miRNAs		Differentially expressed miRNAs
	Control	Mixture of EDCs	
Precursor miRNA	540	534	4 (0.75%)
Mature miRNA	772	761	10 (1.31%)
IsomiRs	3552	3434	36 (1.05%)

The boxes display the number of different sequences found with sncRNA-Seq designed from RNA libraries of pools of control and exposed mouse testes (n = 3). The final row displays the number (percentage) of differentially expressed miRNA: miRNA-precursors, canonical miRNA, and isomiRs variants.

Among the differentially expressed miRNAs in control and exposed mice, precursor and mature forms of *miR-34b-5p* were up-regulated in EDC-exposed animals (Table 2). *miR-34b-5p* is involved in the regulation of genes relevant for cell cycle control, apoptosis, and infertility^23, 24^. Thus, it could explain the increased cell death observed in the testes of mice exposed to the EDCs mixture. As for other miRNAs, the most up-regulated was *miR-7686-5p*, which has no validated mRNA targets, and eight others were down-regulated (Table 2).

**Table 2:** List of miRNA populations differentially expressed after EDCs exposure.

ID	Control	Mixture of EDCs	Fold-change (log_2_)	Differential expression
	% of total identified reads	% of total identified reads		
*pre-mir-34b* (*)	2.3903	3.7084	0.5025	Up
*pre-mir-15b* (*)	0.2256	0.1676	−0.5599	Down
*pre-mir-1291* (*)	0.0052	0.0020	−1.5419	Down
*pre-mir-378b* (*)	0.0049	0.0017	−1.6123	Down
*miR-7686-5p*	0.0007	0.0038	2.3565	Up
*miR-34b-5p* (*)	1.8976	3.1490	0.5968	Up
*miR-18a-5p*	0.0371	0.0277	−0.5548	Down
*miR-15b-5p* (*)	0.2152	0.1596	−0.5656	Down
*miR-1981-5p*	0.0578	0.0311	−1.0274	Down
*miR-382-5p*	0.0049	0.0023	−1.2120	Down
*miR-20b-5p*	0.0042	0.0019	−1.2874	Down
*miR-1291* (*)	0.0047	0.0019	−1.4509	Down
*miR-378b* (*)	0.0047	0.0017	−1.6208	Down
*miR-3085-3p*	0.0024	0.0004	−2.6208	Down

Table shows differentially expressed testicular precursors and canonical miRNAs in control and chronically exposed animals to the mixture of EDCs. (*) The asterisk indicates increase or decrease in both the precursor and the mature form of the miRNA. RNA libraries of pools of mice testes, fold change (log_2_). Data normalised using the DeSeq tool of the R/Bioconductor software package, n = 3, *p ≤ 0.05.

Some miRNAs are expressed in a polycistronic-like form, which means that they are derived from a single loci and grouped in families and clusters^25, 26^. An important miRNA family involved in testis development and physiology is *miR-17*, which is included in the *miR-17-92* cluster with two identified paralogs: the *miR-106a~363* and *mir-106b-25* clusters. When we searched in the sncRNA-Seq data for any member of this *miR-17* family that was differentially expressed in testes after exposure to the mixture of EDCs, we only identified two down-regulated miRNAs: *miR-18a-5p* and *miR-20b-5p*. For the other members of the family, we did not find differences between control and exposed mice (see Supplementary Fig. S2). This result suggested that, while they originated from the same precursor, post-transcriptional regulatory events could explain the differences of expression in control and exposed mice.

**Figure 5:**
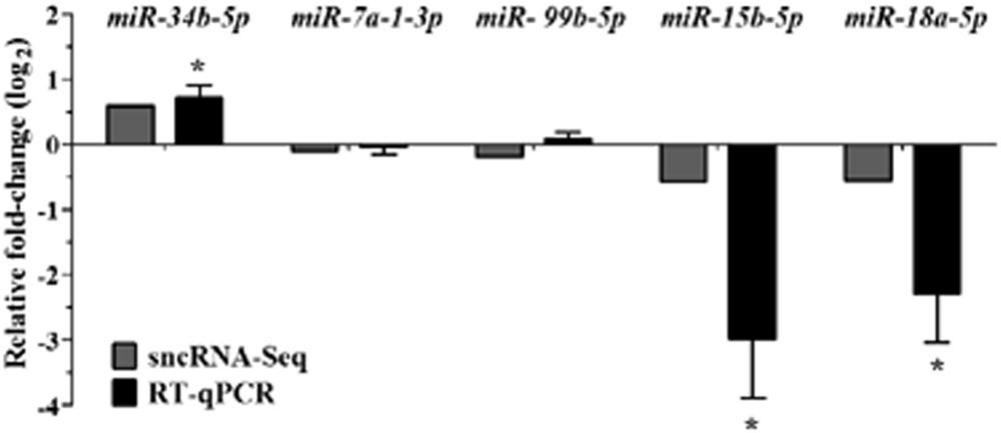
Validation of differentially expressed miRNAs. SncRNA-Seq (gray bars) and RT-qPCR (black bars) of selected mature miRNAs expression in mice testes exposed to the mixture of EDCs vs control mice. DESeq normalised values for sncRNA-Seq and fold change (log_2_) for both sncRNA-Seq and TaqMan RT-qPCR in relation with miRNA expression of control mice ± SEM, n = 4, Mann–Whitney U test, *p < 0.05.

Afterwards, we validated our data via RT-qPCR with five different miRNAs: three differentially expressed and two unchanged. We selected *miR-34b-5p* since its precursor and mature form were up-regulated and it had been implicated in apoptosis, a process that was seen to increase after exposure to the mixture of EDCs. The two others were *miR-15b-5p* and *miR-18a-5*, miRNAs that had been implicated in spermatogenesis and sperm function^27, 28^ and whose precursors and mature forms were down-regulated. We also selected *miR-7a-1-3p* and *miR-99b-5p* as controls, as their levels did not vary in control and exposed mice. The results of RT-qPCR showed that *miR-34b-5p*, *miR-7a-1-3p*, and *miR-99b-5p* levels were similar to those found using sncRNA-Seq. On the other hand, changes observed in *miR-18a-5p* and *miR-15b-5p* were higher with RT-qPCR than with NGS, but the tendency was similar to that observed with sncRNA-Seq (Fig. 5). *miR-7a-1-3p* and *miR-99b-5p* levels were similar to those found with sncRNA-Seq.

**Figure 6:**
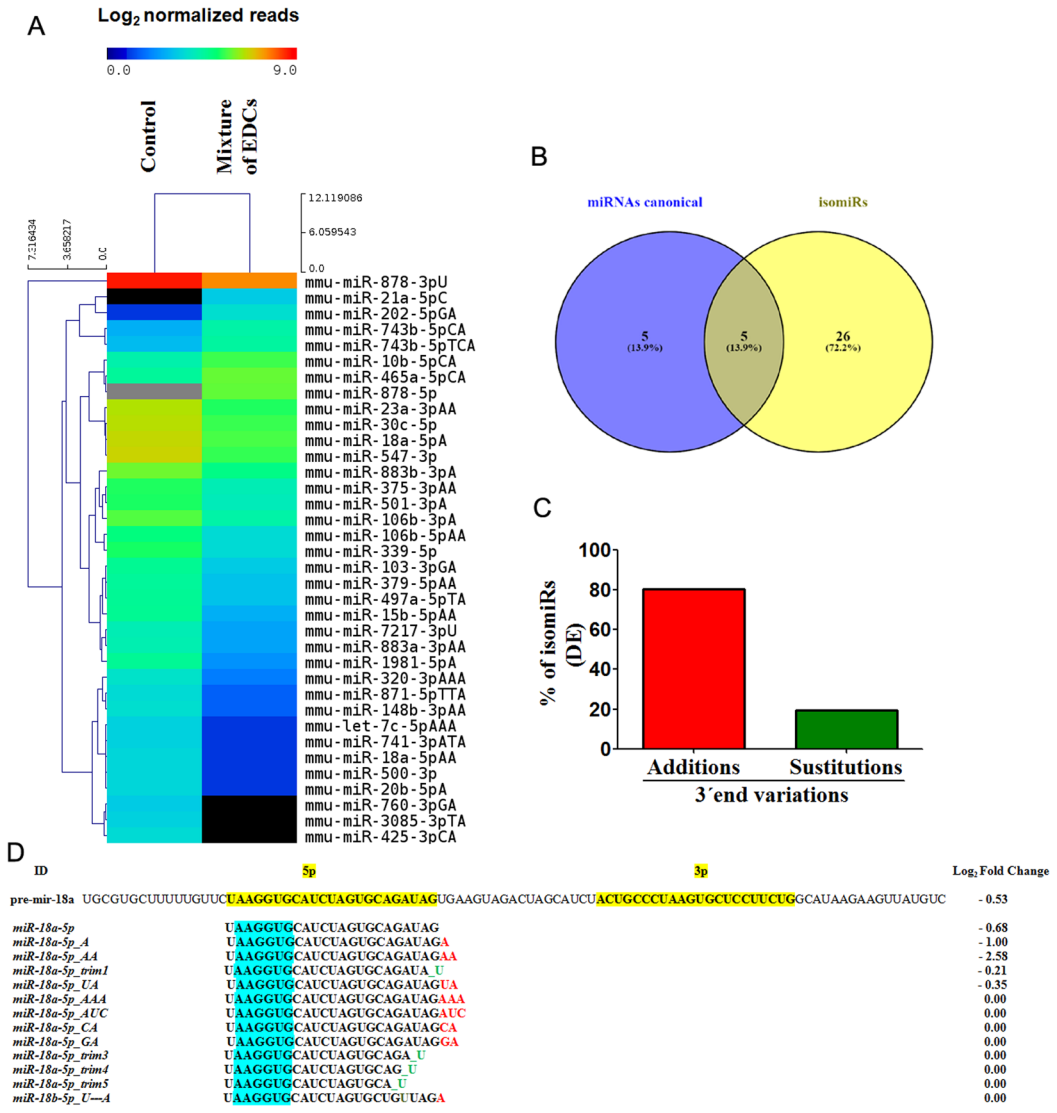
Analysis of differentially expressed isomiRs. (**A**) Hierarchical cluster analysis of differentially expressed isomiRs. Expression levels correspond to log_2_ normalised read counts using DeSeq tool of the R/Bioconductor software package, in testes of control mice and mice exposed to the mixture of EDCs. (**B**) Venn diagram showing the number of differentially expressed isomiRs in relation to canonical miRNAs. (**C**) Modifications of differentially expressed isomiRs variants, affecting the 3′ end. No variants were detected at the 5′ end. (**D**) Sequences and values of expression levels compared to control condition for normalised reads of *pre-mir-18a-5p*, canonical *miR-18a-5p* and *miR-18a-5p*-derived isomiRs. Fold change (log_2_). Data normalised using DeSeq tool of the R/Bioconductor software package, *p ≤ 0.05.

In the present study, we identified isomiRs that were differentially expressed in mice exposed to the EDCs mixture. Using IsomiRage software, we detected 3500 isomiRs in adult mouse testes, and 36 (1.05%) presented differential expression (Table 2). These included four up-regulated and 32 down-regulated isomiRs (Fig. 6A). The number of differentially expressed isomiRs was four-fold higher than that of canonical miRNAs due to the EDCs-mixture exposure. Interestingly, two isomiR variants of *miR-18a-5p* and one isomiR variant of *miR-15b-5p*, *miR-20b-5p*, *miR-3085-3p*, and *miR-1981-5p* were down-regulated due to the addition of an adenine at its 3′ end, similarly to the corresponding canonical miRNAs (Fig. 6A,B). These results were in concordance with new evidence indicating that isomiRs profiles can distinguish a pathological state from a normal one better than canonical miRNAs^29^. In addition, it is important to note that 80% of the differentially expressed isomiRs sequences are miRNAs variants produced by adenine (A) or uracil (U) nucleotide addition events at their 3′ end. The other 20% experienced 3′ end substitution events. In comparison to the canonical miRNAs, none of the 36 isomiRs had substitutions or additions in their 5′ ends or in their seed regions (Fig. 6C,D). These results could indicate that all these isomiRs had equal targeting properties with respect to the corresponding canonical miRNAs, suggesting that they could work along with canonical miRNAs to regulate target mRNAs during spermatogenesis.

The altered expression profile of miRNAs suggested that hormonal imbalance and histological modifications such as germ cell apoptosis, in testes of exposed mice could be an aftermath of changes in some biological processes that are fine-tune regulated by miRNAs. Therefore, we searched for the most reliable sets of mRNA targets of differentially expressed miRNAs. We found that exposure to the mixture of EDCs induced changes in specific miRNAs, which were involved in the regulation of genes implicated in spermatogenesis (Table 3). This was corroborated using GO annotation, in which detected mRNAs were involved in processes such as: hormonal signalling, genitalia development, cell proliferation, programmed cell death, histone H3-K4 trimethylation, protein folding, RNA polymerase transcription factor activity, and phosphatidylinositol phosphatase activity (see Supplementary Table S1).

**Table 3:** Differentially expressed miRNAs are associated with selected target genes in testes of mice exposed to the mixture of EDCs.

miRNA expression	mRNA target (predicted expression)	Function	Involved in	References
*miR34b-5p* (Up)	*Birc5* (Down)	Antiapoptotic protein	Germ cell apoptosis	76
*miR34b-5p* (Up)	*Axl* (Down)	Tyrosine kinase receptor	Germ cell apoptosis	77
*miR34b-5p* (Up)	*Yy1* (Down)	Transcription factor	Germ cell apoptosis	78
*miR34b-5p* (Up)	*Bcl-2* (Down)	Antiapoptotic protein	Germ cell apoptosis	79, 80
*miR34b-5p* (Up)	*Sirt1* (Down)	Intracellular regulatory protein/deacetylase activity	Apoptosis/Spermatogenesis and hormonal disruption	81, 82
*miR34b-5p* (Up)	*Pparγ* (Down)	Nuclear receptor	Hormonal status	83, 84
*miR-34b-5p, miR-7686-5p* (Up)	*Lgr4* (Down)	G-protein-coupled receptors	Apoptosis/Spermatogenesis disruption	85
*miR-34b-5p, miR-7686-5p* (Up)	*Foxj2* (Down)	Transcription factor	Apoptosis/Spermatogenesis disruption	23, 86
*miR-34b-5p, miR-7686-5p* (Up)	*Mllt3* (Down)	Transcription factor	Apoptosis/Spermatogenesis disruption	87
*miR-18a-5p* (Down)	*Nr1h2* (Up)	Transcription factor	Hormonal status	65, 67
*miR-18a-5p, miR-20b-5p, miR-15b-5p, miR-1981-5p, miR-382-5p* (Down)	*Star* (Up)	Cholesterol transporter	Hormonal status	88, 89
*miR-18a-5p* (Down)	*Hsf1* (Up)	Transcription factor	Apoptosis/Spermatogenesis disruption	90
*miR-18a-5p* (Down)	*Pten* (Up)	Phosphatase	Apoptosis/Spermatogenesis disruption	91
*miR-18a-5p, miR-20b-5p* (Down)	*Nfat5* (Up)	Transcription factor	Germ cell apoptosis	92
*miR-15b-5p* (Down)	*Stat3* (Up)	Transcription factor	Germ cell apoptosis	93
*miR-15b-5p, miR-1981-5p* (Down)	*Strbp* (Up)	Poly(A) RNA-binding	Spermatogenesis disruption	94
*miR-15b-5p, miR-20b-5p* (Down)	*Ccnd2* (Up)	Regulators of CDK kinases	Germ cell apoptosis	95, 96
*miR-15b-5p, miR-20b-5p* (Down)	*Ccnd1* (Up)	Regulators of CDK kinases	Germ cell apoptosis	93
*miR-15b-5p, miR-20b-5p* (Down)	*Atg9a* (Up)	Autophagy	Germ cell apoptosis	97
*miR-20b-5p* (Down)	*Itgb8* (Up)	Adherens junctions	Testis injury	98
*miR-20b-5p* (Down)	*Ccne1* (Up)	Regulators of CDK kinases	Germ cell apoptosis	99
*miR-20b-5p* (Down)	*Bambi* (Up)	Tgfβ inhibitor	Spermatogenesis disruption	100
*miR-382-5p* (Down)	*Capn8* (Up)	Calpain inhibitors	Germ cell apoptosis	101, 102
*miR-382-5p* (Down)	*Vim* (Up)	Cytoskeleton	Hormonal status	103
*miR-1291* (Down)	*Dnmt3a - Dnmt3b* (Up)	DNA methylation	Testis injury, transgenerational inherence, testis cancer	57, 58

Target predictions of miRNAs were done using IPA and miRWalk. Function and biological process involvement in the testes for each target gene were recorded from references in the literature.

### *miR-18a-5p* was associated with the reduction of intratesticular estradiol levels in testes of mice exposed to EDCs mixture

**Figure 7:**
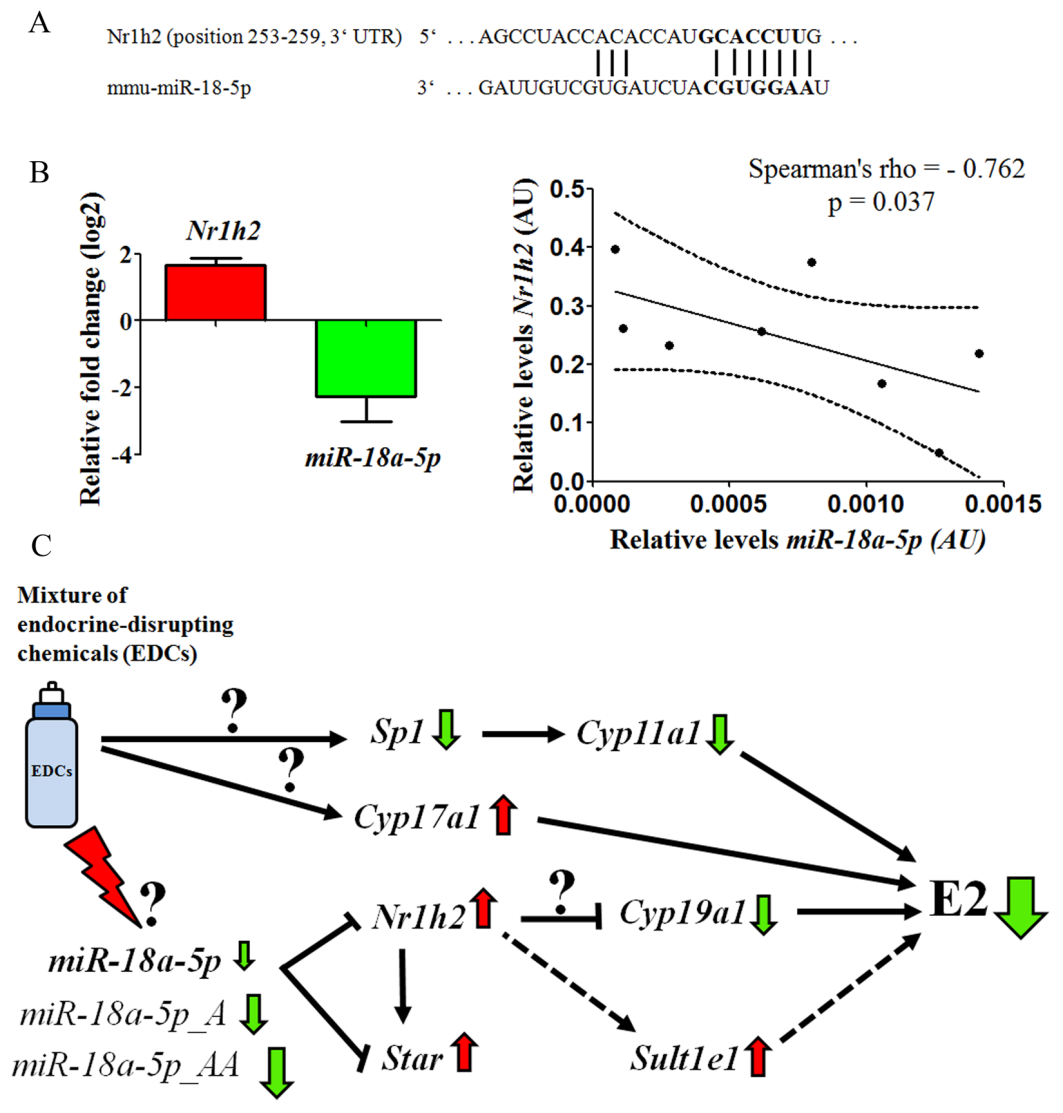
Down-regulation of miR-18a-5p in mice exposed to EDCs mixture is associated with estradiol decrease in testes via *Nr1h2*. (**A**) Prediction of *Nr1h2* as a target of *miR-18a-5p*, (**B**) mRNA expression of *Nr1h2* and *miR-18a-5p* in the testes of mice exposed to EDCs mixture versus control mice by RT-qPCR, fold change (log_2_) ± SEM, n = 4, and *Nr1h2*:*miR-18a-5p* Spearman’s rank correlation coefficient (Spearman’s rho), *p < 0.05. Abbreviation: AU, arbitrary units. (**C**) Role of *miR-18a-5p* in the estradiol decrease in the testes of mice exposed to EDCs mixture (proposed model). Red arrow = up-regulated, green arrow = down-regulated.

Giving that the exposure to a mixture of EDCs induced a decrease in estradiol levels, we correlated the differentially expressed miRNAs with deregulated transcripts of the steroidogenic pathway. We used bioinformatics tools and found that in mouse testes, *Nr1h2* could be a target of *miR-18a-5p* (Fig. 7A). To support our prediction, we searched in the DIANA-TarBase v7.0, that contains hundreds of thousands of high-quality manually curated and experimentally validated miRNA:gene interactions^30^ for *miR-18a*. We found that in the mouse, targeting of *Nr1h2* by *miR-18a-5p* was already validated using immunoprecipitation experiments with Ago2 in C1C12 cells^31^.

The nuclear receptor subfamily 1, group H, member 2 (*Nr1h2*), is a positive transcriptional factor for *Star, Cyp11a1* and *Hsd3b1* and might act as a negative regulator of *Cyp19a1* expression to control steroidogenesis^32, 33^. By RT-qPCR, we found that *Nr1h2* levels were two-fold higher in testes of mice exposed to EDCs mixture, which were negatively correlated with *miR-18a-5p* (Fig. 7A,B), suggesting a mechanism of estradiol downturn by various pathways and associated with loss of *miR-18a-5p* induced by the exposure to the EDCs mixture (Fig. 7C).

In conclusion, chronic exposure to a mixture of five EDCs induces changes in the expression profiles of specific miRNAs (such as *miR-34b-5p, miR-7686-5p* and *miR-1291*), along with alterations in the miRNAs/isomiRs association (in particular for *miR-15b-5p, miR-18b-5p, miR-20b-5p*, and *miR-1981-5p*) regulating mRNAs implicated in key biological process in the testes (Table 3).

## Discussion

In the present study, we demonstrated for the first time in mice that a chronic low-dose exposure to a mixture of phthalates and alkylphenols, from an early embryo stage to adulthood, induces alterations in testes which are associated with deregulation of miRNAs and isomiRs expression, along with a deregulation of genes that encode proteins involved in the biogenesis, editing, and stability/degradation of these sncRNAs.

Of interest, was the presence of foci or regions of Leydig cells displaying hyperplasia (although the hyperplasia of these cells could not be quantified) (Fig. 2) as in humans it is associated with disturbances in spermatogenesis, germ cell tumours, oligozoospermia and azoospermia^34, 35^.

In this work, we showed that a mixture of three phthalates and two alkylphenols acts as an endocrine disruptor inducing decrease in the intratesticular estradiol levels in exposed mice. This was correlated with changes in the expression of genes that regulate spermatogenesis which include *Sp1*, *Star* and steroidogenesis-pathway enzymes in testes, especially the early down-regulation of aromatase (*Cyp19al*) in neonatal and adult mice (Fig. 3). Although an exposure to the mixture of EDCs did not alter testosterone in serum nor intratesticular fluid (Fig. 3, see Supplementary Fig. S3), it correlates with recent studies *in vivo* where repeated doses of phthalates did not affect testosterone production^36^. In addition, it is possible that the co-administration of some EDCs antagonizes the increase of testosterone stimulated by other EDCs^7^.

Interestingly, serum estradiol was found unchanged (see Supplementary Fig. S3), which suggests a compensatory mechanism mediated by the synthesis of estradiol in adipose tissue, which is associated with the increase of body weight in relation to reports of obesogenic effects due to an exposure to EDCs^37^. Furthermore, the decrease of aromatase and estradiol levels but not of androgens in adult mouse testes could be related to Leydig cell hyperplasia, seminiferous tubular injury and germ cell apoptosis, in a similar way reported in the ArKO model^38^. All of these are clear indicators of reproductive pathology and good evidence of the risk that chronic exposure to mixtures of EDCs poses.

Previous works demonstrated that altered testes phenotypes due to a single EDCs exposure were the result of global changes in the transcriptome^6^. New research has proposed a link between these changes in mRNA expression and the loss of the post-transcriptional control that miRNAs or other sncRNAs mediate. Our work found evidence for deregulation of mRNA levels of several components of the miRNAs biogenesis machinery at the level of pri-miRNA processing, editing and stability/degradation of pre-miRNAs (Fig. 4). These results suggest that chronic exposure to a mixture of EDCs induces changes in the global activity of miRNAs.

However, our data revealed that exposure to a mixture of EDCs resulted in the deregulation of specific miRNAs and isomiRs with 3′ end variants, some of them of differentially expressed canonical miRNAs (as shown for *miR-18a-5p*) (Fig. 6) which, consequently, share mRNA targets. These isomiRs could function in a cooperative manner with the corresponding canonical miRNAs to target the same mRNAs^39, 40^. Interestingly, this supports the notion that the differential expression of isomiRs is not a random event. Selective miRNAs:mRNAs interaction could be related to the functional stability of isomiRs with respect to canonical miRNAs^41, 42^. This phenomenon could be associated with cellular response to stress (which the EDCs mixture induces in this case), facilitating more stable isomiRs or canonical miRNAs and modifying the miRNA biogenesis programs, including Dicer1 isoforms^43^ and miRNA nucleotidyl transferases such as *PAPD4*, *PAPD5*, and *ZCCHC11* ^44^. To support this hypothesis, we detected over-expression of *Zcchc11* mRNA, which is implicated in isomiRs generation by uridylation. Alterations in isomiRs levels, together with deregulation of the enzymes involved in their generation, suggest that exposure to EDCs deregulates miRNAs turnover. It would be ideal to undertake further studies concerning deregulation of isomiRs variants induced by EDCs or other environmental toxicants.

Regarding canonical miRNAs, our sncRNA-Seq data showed changes in the expression of 10 mature miRNAs in the testes of exposed mice (Table 2) and some of them were validated by RT-qPCR (Fig. 5). One of them, *miR-34b-5p*, was abundantly expressed in the testes. Previous reports revealed that *miR-34b/c* deficiency is correlated with oligoasthenoteratozoospermia and infertility in mice^23, 24^ along with “Sertoli cell only” syndrome, mixed atrophy, and arresting of germ cell differentiation in the biopsies of infertile patients^45, 46^. Hence, we suggest an important relationship between exposure to mixtures of EDCs and testicular damage due to changes in the expression of miRNAs, such as *miR-34b-5p*. This suggests that miRNAs are good biomarkers of reproductive pathologies induced by exposure to environmental contaminants. Furthermore, *miR-34b* is an important factor in the maintenance of spermatogenesis as it is necessary to the control of post-mitotic germ cell development and apoptosis. The luciferase-based reporter assay and ChIP showed that protein p53 induces transcriptional activation by binding to the promoter of *pri-mir-34* ^47, 48^. In addition, p53 facilitates Drosha and the microprocessor complex processing of pri-miRNA into pre-miRNA^49^. Once activated, *miR-34b* and p53 may contribute directly to the regulation of apoptosis, cell cycle, and proliferative gene targets^50^. In return, some validated targets of *miR-34b* are anti-apoptotic factors and post-translational p53 inhibitors, which indicate that *miR-34b* can stabilize p53 in response to genotoxic stress^51^. These reports and our *in silico* analysis (Table 3) indicate that *miR-34b-5p* could be a pro-apoptotic factor induced in germ cells after EDCs exposure. Thus, it is possible that the up-regulation of *miR-34b-5p* explains in part the increase of germ cell apoptosis that we observe in exposed testes.

Besides *miR-34b-5p*, we found that *miR-7686-5p* levels were significantly up-regulated in exposed mice (Table 2). There were no previous studies regarding the function of this miRNA, but our bioinformatics analysis predicted that its mRNA targets could be associated with germ cell apoptosis and spermatogenesis disruption (Table 3).

On the other hand, we found eight down-regulated miRNAs, some of them implicated in multiple processes such as cancer (*miR20b-5p*, *miR-1291*)^52, 53^, organ injury in toxicity drug models (*miR-382-5p*)^54^, metabolic processes and steroidogenesis (*miR-378b*)^55^, and tissue inflammation (*miR-3085-3p*)^56^. None of them has been studied in the context of testes physiology. However, when we analysed the predicted targets and GO process, we found that *miR-1291* (miRNA with unknown role in the testis) had as target DNA methyltransferases (*Dnmt3a*, *Dnmt3b*) (Table 3) involved in *de novo* histone methylation, genomic imprinting, X-chromosome inactivation and testicular germ cell tumours due to exposure to alkylphenols^57, 58^. This suggests that the down-regulation of sncRNAs such as *miR-1291* due to exposure to an EDCs-mixture might promote changes in the DNA methylation pattern related to the epigenetic transmission of adverse effects^59^.

Interestingly, the targets of the down-regulated *miR-15b-5p* in exposed mice, for instance, *Ccnd2*, *Ccnd1*, and *Raf1* (Table 3), are implicated in cell cycle regulation as well as cell survival and cancer^60^. Moreover, in a Sertoli cell line, researchers detected that NP induces a decrease of *miR-15b* ^19^. Our sncRNA-Seq data and RT-qPCR showed that *miR-15b-5p* levels decreased *in vivo* in the testes of mice exposed to the mixture of EDCs containing NP. Research also demonstrated down-regulation of this miRNA in biopsies of infertile men with “Sertoli cell only” syndrome^45^. The suggestion that *miR-15b-5p* could be involved in the control of spermatogenesis through Sertoli cells and in testes pathologies related to the exposure to EDCs is worthy of assessment in further studies.

Another important miRNA that was down-regulated by EDCs-mixture was *miR-18a-5p*, which is also associated with Sertoli cells; its absence results in the deterioration of spermatogenesis^61^. *Hsf2* - a transcriptional factor of genes that is required for successful spermatogenesis - and *Pten -* which is implicated in apoptosis induced by EDCs - are validated mRNA targets of this miRNA^26, 62^. Interestingly, these miRNAs belong to the cluster, *miR-17-92*, which is expressed in a polycistronic manner. However, we observed that *miR-18a-5p* was the only deregulated miRNA from this cluster (see Supplementary Fig. S2). As the processing of *pre-mir-18a* stem-loop may be selective and independent of the cluster by the action of RNA-binding proteins^63, 64^, we speculate that exposure to the mixture of EDCs could alter the expression of some RNA-binding proteins, affecting the processing of specific miRNAs.

Despite not having determined, in this work, the participation of nuclear receptors that are known to interact with the EDCs used such as ER, RXR/PPAR and AhR, we found that the decrease of *miR-18a* is negatively correlated to the *Nr1h2* levels detected in exposed mice. This transcriptional factor is expressed in Sertoli and germ cells, and was found deregulated in testicular biopsies of patients with azoospermia^65^. Consistently, its ligands (sterols) have been up-regulated in the case of oxidative stress and cell death^66^. Although the mechanism is still unknown, there is evidence that exposure to phthalates, alkylphenols and others reprotoxicants such as radioactive elements that interfere with the expression of genes involved in the steroidogenesis, increase *Nr1h2* levels^67, 68^. Furthermore, new evidence suggests that exposure to a mixture of the phytoestrogen Genistein and DEHP in human-exposure corresponding doses may be also involved in the *Nr1h2* agonism^69^. Here we suggest that up-regulation of *Nr1h2* might be due to a down-regulation of *miR-18a-5p* (and potentially its isomiRs) and it may be part of a common mechanism for changing the expression of steroidogenic-pathway transcripts such as *Star* and *Cyp19a*1 (or estrogen sulfotransferase) that are involved in the decrease of estradiol levels (Fig. 7). Therefore, future studies should be performed on isolated testicular cells and *in vitro* systems to validate miRNAs:mRNAs interactions shown in this work.

In conclusion, the present study shows that a deregulation of a small group of miRNAs and isomiRs in male mice chronically exposed to a low-dose of an environmental mixture of EDCs would have consequences on mRNA targets and testicular physiology. These changes would suffice to trigger the phenotype of testes injury characterised by a decrease of intratesticular estradiol levels, spermatogenesis disruption, germ cells apoptosis and could be involved in male infertility.

## Material and Methods

### Animals and ethical statement

We carried out all procedures relating to the care and handling of animals in accordance with the regulations of the CSIC and the Catholic University of Chile (UC), following the European Commission (EC) guidelines (directive 86/609/EEC), and the guides of the National Research Council of Chile, respectively. The General Direction of Environment of CAM in Spain (Ref. PROEX 054/15) and the National Fund of Science and Technology (FONDECYT) (No. 1150532) in Chile reviewed and approved all the experimental protocols in this work. C57BL/6J mice were bred at the CSIC or UC bioterium under specific, pathogen-free (SPF), temperature-controlled and humidity-controlled conditions in a 12-hour light/dark cycle with *ad libitum* access to food and water.

### Chemicals

Bis (2-ethylhexyl) phthalate (DEHP), dibutyl phthalate (DBP), benzyl butyl phthalate (BBP), 4-nonylphenol (NP), 4-tert-octylphenol (OP) and DMSO (dimethyl sulfoxide) were purchased from Sigma-Aldrich Co, (USA). Ethanol was acquired from Winkler (Chile).

### Exposure to an endocrine-disrupting mixture

We designed a defined mixture (bulk stock) that contained three phthalates [(DEHP), (DBP), and (BBP)] diluted in DMSO and two alkylphenols [(NP) and (OP)] diluted in ethanol. We dissolved and administered the mixture in the drinking water of C57BL/6J mice with *ad libitum* access to food and water. We calculated the final dose according to the volume of water ingested by the mice and the body weight was recorded in a pilot study in agreement with the literature referring to these parameters.

Moreover, pilot studies were designed in order to evaluate the effect of three different dose of the mixture of EDCs, relative to LOAEL (Lowest Observed Adverse Effect Level) dose for each compounds. To this end, we treated pregnant females and evaluated the number of pups after chronic exposure during the pregnancy period. The highest dose (30 mg/kg-BW/day of each phthalate and 5 mg/kg-BW/day of each alkylphenol) was considered toxic since it decreased the number of neonatal mice whereas the medium dose (3 mg/kg-BW/day of each phthalate and 0.5 mg/kg-BW/day of each alkylphenol) presented no change of the hormonal status in the testes of male offspring (see Supplementary Fig. S4). We decided to use an environmentally relevant low-dose exposure of 0.3 mg/kg-BW/day for each phthalate and 0.05 mg/kg-BW/day for each alkylphenol. As for the control group, the water was supplemented with an equivalent dose of vehicle with DMSO at an estimated intake of 0.25 g/kg-BW/day and ethanol of 0.06 g/kg-BW/day. Considering the multiple studies to assess the LOAEL for the various compounds used in this experimental mixture, the dosages used in the present work were at least ~1,000-fold lower than the LOAEL values for reproductive effects in male animals.

To emulate chronic human exposure to an environmental mixture of EDCs, we administered the mixture of EDCs or vehicle (control) to pregnant mice from post-coital day 0.5 (conception), throughout gestation, childbirth, and lactation. At weaning, we selected the male offspring and maintained a maximum of four male mice with the mother per cage. We continued the administration of the mixture of EDCs or control until adulthood (endpoint: postnatal day 60) (Fig. 1).

In all experiments pregnant mice randomly selected on post-coital day 0.5 were defined as (n), with a minimum of three pregnant mice treated per group. Male offspring per (n) of each group were considered biological replicates.

### Testes histology

At the endpoint, we determined the body and testis relative weight for each mouse, then fixed one testis in Bouin solution, embedded it in paraffin, and assessed sections of 7 μm mounted on slides through PAS staining (periodic acid-Schiff, counterstained with hematoxylin). Histology was evaluated using an Olympus CX31 microscope (Olympus, Japan) and apoptosis in the histological sections was quantified by its pyknotic appearance (100 seminiferous tubules per each replicate, per (n)). Moreover, pictures were taken with a 5XC-3 digital camera (Olympus, Japan) and morphometric analyses performed with Image J software.

### Immunohistochemistry

Sodium citrate buffer (0.01 M and pH 6) was used on testis sections with an antigen retrieval step. UltraVision detection system (Thermo-Scientific, USA) was applied in immunohistochemistry assays, as previously described by Urriola-Muñoz, P. *et al*.^22^. Slides were incubated overnight at 4 °C with an anti-caspase 3 antibody (Cell Signaling, USA) at 1 mg/ml and the sections counter-stained with hematoxylin and subsequently evaluated under a microscope. Active caspase-3 cells were quantified in a minimum of 100 seminiferous tubules per each replicate, per (n).

### Intratesticular hormone analyses

Seminiferous tubular fluid (STF) was isolated from whole testes according to Jarow *et al*.^70^. For each testis per animal, we diluted 5 µl of STF in 300 µl of PBS and assessed testosterone and estradiol levels by radioimmunoassay (RIA). All samples were assayed simultaneously and run in duplicate. Sensitivity, intra- and inter-assay coefficients of variation (CV) for testosterone were 0.01 pg/µl, CV < 13.4% and <7.6%, respectively, and those for estradiol were 0.05 pg/µl, CV < 11.3% and <24.9%, respectively.

### RNA Isolation

RNA was isolated from decapsulated testes using TRIzol® Reagent, its concentration verified with a NanoDrop ND-1000 spectrophotometer (NanoDrop) and the integrity determined on a 2100 Bioanalyzer (Agilent, USA), accepting a RIN > 7.

### Expression of mRNA by RT-qPCR

We performed retrotranscription (RT) to cDNA using 500 ng of total RNA with Oligo dT17, 1X first-strand buffer (Invitrogen), 0.01 M dithiothreitol (DTT), 0.1 mM of each dNTP, and 200 U of superscript II (Invitrogen, USA). RT-qPCR reactions were carried out with 1 μl of cDNA and 0.0625 μM of each specific primer in a 20 μl reaction volume using thermal cycles according to García-López, J. & del Mazo, J.^71^, for primer sequence (see Supplementary Table S2). Data was normalised using the 2^−ΔΔCt^ method^72^ with *Gapdh*, *H2afz*, and *Ppia* as endogenous reference genes, and following the MIQE guidelines^73^.

### Amplification of mature miRNAs

Hydrolysis probes PCR-primer-TaqMan® system (Applied Biosystems, USA) was used to analyse the mature miRNA forms. In short, we retro-transcribed the RNA isolated from testes containing the miRNA fraction, into cDNA using specific stem–loop reverse transcription primers in accordance with the manufacturer’s recommendations (Applied Biosystems, USA). After cDNA conversion, we carried out RT-qPCR using specific TaqMan® real-time PCR primers and a 7500 fast real-time detection system (Applied Biosystems, USA). A 20 μL PCR reaction volume contained 2 μL of RT products, 1 × TaqMan® Universal PCR master mix, 1 μL of primers and the probe mix of TaqMan® MicroRNA assay kit (Applied Biosystems, USA). Then, reactions were incubated at 95 °C for 10 minutes, followed by 40 cycles at 95 °C for 15 seconds and 60 °C for 1 minute. Subsequently, expression levels of mature miRNAs were evaluated using the 2^−ΔΔCt^ method^72^. Transcription level of U6 was used as a reference endogenous gene.

### Small non-coding RNA sequencing (sncRNA-Seq)

For small RNA-seq library generation, we prepared 1.5 µg of RNA (RIN > 9) isolated from a pool of testes of control and exposed to the mixture of EDCs mice [(n = 3, equal amounts of RNA per (n)], according to the Illumina protocols (www.illumina.com/support). We performed small RNA-seq using MiSeq Sequencing System (Illumina, USA) in the single-end mode with a read length of 65 nucleotides. Then, we carried out the quality control of raw read data using the FastQC program (http://www.bioinformatics.babraham.ac.uk/projects/fastqc/) and Cutadapt software to trim off the adapters. Reads shorter than 12 nucleotides were removed. Trimmed reads were aligned with the mouse genome (10 mm) using bowtie, allowing up to three mismatches in v mode (-y –strata –best -a –chunkmbs 256). Afterwards, we undertook the identification and quantification of pre-miRNAs and mature miRNAs using HTSeq script^74^ and a general feature format file (GFF) that was downloaded from miRBase v21. IsomiRage software was used for isomiRs detection and quantification^75^.

### Identification of miRNA targets and functional analysis

We targeted differentially expressed miRNAs and isomiRs using the miRWalk database, combining the searches of several databases with the validated mRNA targets and DIANA-TarBase v7.0. In addition, we used Ingenuity Pathway Analysis software (IPA) to obtain the mRNAs targets.

Functional analysis for potential mRNAs targets in biological processes and molecular function of GO domains were performed using a Cytoscape plugging ClueGO and CluePedia. We also carried out gene ontology enrichment analysis using a hypergeometric test with a p-value threshold less than 0.05.

### Data deposition

Public database accession is provided for all raw data sets and processing data. These have been deposited in the NCBI Gene Expression Omnibus (GEO); GSE84695 (http://www.ncbi.nlm.nih.gov/geo/query/acc.cgi?acc=GSE84695).

### Statistical analysis

We carried out data and gene expression analysis by RT-qPCR using GraphPad Prim version 5.0. Differences in the averages observed with unpaired t test and Mann-Whitney U test were analysed. Comparisons between different mixtures were carried out using one-way analysis of variance (ANOVA) followed by Dunnett´s *post hoc* test. In addition, we carried out data normalisation and differential expression analysis of sncRNAs-Seq using the DESeq tool of the R/Bioconductor software package. In all analyses, differences with a p-value less than or equal to 0.05 were considered significant.

## Electronic supplementary material

Supplementary information
