# Supplementary material for: Chronic low-dose exposure to a mixture of environmental endocrine disruptors induces microRNAs/isomiRs deregulation in mouse concomitant with intratesticular estradiol reduction
Source: Sci Rep. 2017 Jun 13;7:3373. doi: 10.1038/s41598-017-02752-7 (PMC5469815; doi:10.1038/s41598-017-02752-7)
Supplement: Supplementary file 1 — Supplementary information [file 41598_2017_2752_MOESM1_ESM.doc]

**Chronic low-dose exposure to a mixture of environmental endocrine disruptors induces microRNAs/isomiRs deregulation in mouse concomitant with intratesticular estradiol reduction**

Julio Buñay ^1^, Eduardo Larriba ^2^, Ricardo D. Moreno ^1,*^ and Jesús del Mazo ^2,*^

^1^  Department of Physiology, Pontificia Universidad Católica de Chile, Santiago, Chile.

^2^ Department of Cellular and Molecular Biology, Centro de Investigaciones Biológicas (CSIC), Madrid, Spain.

**Supplementary Info File**

**Supplementary table legends:**

**Supplementary Table S1:** **GO enrichment analysis of potential mRNA targets of differentially expressed miRNAs.**

GO: enrichment was performed using *ClueGO* and obtained by hypergeometric distribution test, *** p-value threshold less than 0.05.

**Supplementary Table S2: Primers used for RT-qPCR**

**Supplementary figure legends:**

**Supplementary Figure S1:** **Testis injury in mice exposed to the mixture of EDCs.**

A) Quantification of seminiferous tubules with germ cells exfoliated towards the tubular lumen. B) Seminiferous tubules without lumina. C) Frequency of seminiferous epithelium cycles. All graphics represent the mean ± SEM. Comparison between control mice and mice exposed to mixture of EDCs; quantification of 100 seminiferous tubules per each replicate per (n). Mann-Whitney U test, n = 4, * p < 0.05, ** p < 0.01.

**Supplementary Figure S2:** **Dendrogram representing hierarchical clustering analysis of *miR-17* family in the sncRNA-Seq libraries.**

Hierarchical cluster analysis of *miR-17* family in testes of mice exposed to the mixture of EDCs comparing to the control mice; sncRNA-Seq. Expression levels correspond to log_2_ normalised read counts using DeSeq tool of the R/Bioconductor software package, n = 3, * p ≤ 0.05.

**Supplementary Figure S3:** **Exposure to the mixture of EDCs does not change testosterone and estradiol serum levels.**

Quantification of testosterone and estradiol serum levels by RIA in mice exposed to the mixture of EDCs comparing to the control mice. All graphics represent the mean ± SEM, n = 4, Mann-Whitney U test.

**Supplementary Figure S4: Pilot study for the selection of the dose to be used in the mixture of EDCs.**

A) Quantification of litter size, B) quantification of intratesticular testosterone and estradiol levels by RIA. Control mice and exposed mice to the mixture of EDCs in: high-dose (30 mg/kg-BW/day of each phthalate and 5 mg/kg-BW/day of each alkylphenol), medium-dose (3 mg/kg-BW/day of each phthalate and 0.5 mg/kg-BW/day of each alkylphenol) and low-dose (0.3 mg/kg-BW/day of each phthalate and 0.05 mg/kg-BW/day of each alkylphenol). All graphics represent the mean ± SEM, n = 6, One-way analysis of variance followed by Dunnett´s *post hoc* test, * p < 0.05, ** p < 0.01.

**Supplementary Table S1.**

| **GO analysis of most probable mRNA target of up-regulated miRNAs** | | | |
| --- | --- | --- | --- |
| **GO_Id** | **GO_Term** | **Domain** | **p-value** |
| GO:0033211 | Adiponectin-activated signalling pathway | Process | 2.76E-03 |
| GO:0007064 | Mitotic sister chromatid cohesion | Process | 3.82E-03 |
| GO:0080182 | Histone H3-K4 trimethylation | Process | 3.82E-03 |
| GO:0070208 | Protein heterotrimerisation | Process | 4.61E-03 |
| GO:0070207 | Protein homotrimerisation | Process | 6.49E-03 |
| GO:0061098 | Positive regulation of protein tyrosine kinase activity | Process | 2.66E-03 |
| GO:0010828 | Positive regulation of glucose transport | Process | 2.98E-03 |
| GO:0010623 | Developmental programmed cell death | Process | 3.53E-03 |
| GO:0048806 | Genitalia development | Process | 3.33E-03 |
| GO:0030279 | Negative regulation of ossification | Process | 1.66E-03 |
| GO:0009755 | Hormone-mediated signalling pathway | Process | 2.87E-03 |
| GO:0004879 | RNA polymerase II transcription factor activity | Function | 2.19E-02 |
|  |  |  |  |
| **GO analysis of most probable mRNA target of down-regulated miRNAs** | | | |
| **GO_Id** | **GO_Term** | **Domain** | **p-value** |
| GO:0061419 | Positive regulation of transcription in response to hypoxia | Process | 4.90E-04 |
| GO:0021960 | Anterior commissural morphogenesis | Process | 3.67E-04 |
| GO:0004438 | Phosphatidylinositol-3-phosphatase activity | Function | 1.86E-04 |
| GO:0090394 | Negative regulation of excitatory postsynaptic potential | Process | 4.56E-04 |
| GO:2000644 | Regulation of receptor catabolic process | Process | 4.56E-04 |
| GO:0045906 | Negative regulation of vasoconstriction | Process | 6.27E-04 |
| GO:1902893 | Regulation of pri-miRNA transcription | Process | 6.70E-04 |
| GO:1900745 | Positive regulation of p38MAPK cascade | Process | 8.84E-04 |
| GO:0060749 | Mammary gland alveolus development | Process | 1.88E-03 |
| GO:0051893 | Regulation of focal adhesion assembly | Process | 5.45E-03 |
| GO:0021954 | Central nervous system neuron development | Process | 3.92E-04 |
|  | | | |

**Supplementary Table S2.**

| **Gene** | **Forward** | **Reverse** | **Product length (bp)** |
| --- | --- | --- | --- |
| *Sp1* | TGCCACCATGAGCGACCAAGATCA | TGCTGCTGCTTCGAGTCTGAGAAA | 137 |
| *Star* | CATCAGAGCTGAACACGGTC | CCACCCCTTGAGGTCAATAC | 106 |
| *Cyp11a1* | AAGTATGGCCCCATTTACAGG | TGGGGTCCACGATGTAAACT | 64 |
| *Cyp17a1* | CTCCAGCCTGACAGACATTCTG | TCTCCCACCGTGACAAGGAT | 117 |
| *Hsd3b1* | ACATGGCTCTGGGAGTTATAAGGT | TTAGTGACTGGCAAGGCTTCTG | 129 |
| *Cyp19a1* | GACAGGCACCTTGTGGAAAT | GAGGTTCACGCCACCTACTC | 150 |
| *Nr1h2* | CGCTACAACCACGAGACAGA | TGTTGATGGCGATAAGCAAG | 178 |
| *Drosha* | GGACCATCACGAAGGACACTT | ATGCCCAGTTCCTCTGCTACCT | 87 |
| *Dicer* | GGATGCGATGTGCTATCTGGA | GCACTGCTCCGTGTGCAA | 142 |
| *Xpo5* | GAAGTAGCCCCCTCGTCTGT | AGCAGTGCTGTGCAGACATC | 83 |
| *Ago2* | GCCGTCCTTCCCACTACCAC | GGTATTGACACAGAGCGTGTGC | 121 |
| *Adar 1S* | GGGTCTTGATCGGGGAGA | CTGCCAGAGAGAGGAAGTG | 145 |
| *Lin28b* | GAGTCCAGGATGATTCCAAGA | TGCTCTGACAGTAATGGCACTT | 106 |
| *Zcchc11* | CAGCAAAGAAAGCCACCAGT | AAAAGGCATTCCATCCATCA | 94 |
| *Zcchc6* | CATTAAAAAGGAATGCCCACA | TTCTTTTTGTCTTCATGTAAAAGCAC | 67 |
| *Snd1* | TCTACATCGACTACGGCAACA | GTGCTGAAGGCAGGTGGTA | 73 |
| *Gapdh* | GCTGATGCTCCCATGTTCGTGAT | GTGGTGCAAGAGGCATTGCTGAC | 86 |
| *H2afz* | ACAGCGCAGCCATCCTGGCGTA | TTCCCGATCAGCGATTTGTGGA | 133 |
| *Ppia* | CGCGTCTCCTTCGAGCTGTTTG | TGTAAAGTCACCACCCTGGCACAT | 150 |

**Supplementary Figure S1:** **Testis injury in mice exposed to the mixture of EDCs.**

**
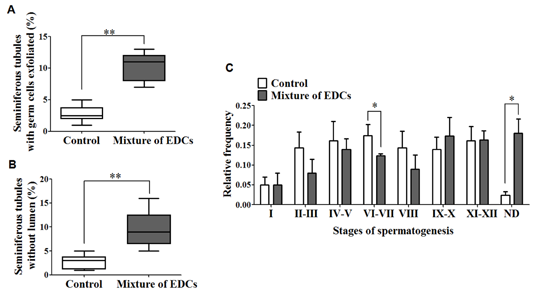
**

**Supplementary Figure S2:** **Dendrogram representing hierarchical clustering analysis of *miR-17* family in the sncRNA-Seq libraries.**

**
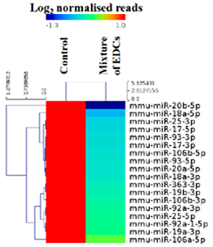
**

**Supplementary Figure S3:** **Exposure to the mixture of EDCs does not change testosterone and estradiol serum levels.**

**
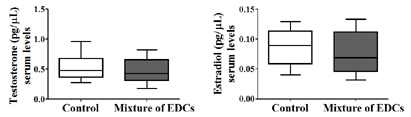
**

**Supplementary Figure S4: Pilot study for the selection of the dose to be used in the mixture of EDCs.**

**
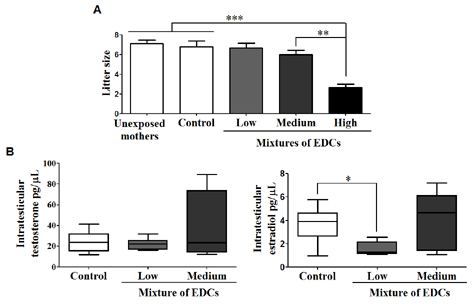
**
